# The effect of 2% chlorhexidine iontophoresis on dentin sealing ability of etch-and-rinse adhesive: An *in vitro* study

**DOI:** 10.1016/j.jds.2023.09.004

**Published:** 2023-09-09

**Authors:** Kanittha Kijsamanmith, Panisara Srisatayasatien, Nichapa Thanindratarn, Chanisa Vichainarong, Jirapat Panyasukum

**Affiliations:** Department of Oral Biology, Faculty of Dentistry, Mahidol University, Bangkok, Thailand

**Keywords:** Chlorhexidine, Etch-and-rinse adhesive, Human dentin, Iontophoresis, Sealing ability

## Abstract

**Background/purpose:**

Iontophoresis could enhance the delivery of chlorhexidine into oral tissue. This study aimed to determine the effect of 2% chlorhexidine iontophoresis (CHI) on the sealing ability of etch-and-rinse adhesive in human dentin using hydraulic conductance (HD) measurement, scanning electron microscopy and energy dispersive x-ray spectroscopy (SEM-EDS).

**Materials and methods:**

Thirty-nine sound dentin specimens were prepared from 39 extracted intact third molars. Thirty specimens were used for HD measurement and randomly divided into 3 equal-sized groups; (1) No chlorhexidine treatment (control), (2) passive chlorhexidine treatment (CHT) and (3) CHI on acid-etched dentin. Each dentin surface was treated with etch-and-rinse adhesive. HD of each specimen was measured before treatment, after immediate bonding and after 14 days. The other 9 specimens were subjected to SEM-EDS analysis of the acid-etched dentin and the dentin treated with CHT and CHI. ANOVA test and Student-Newman-Keuls method were used for statistical analysis.

**Results:**

After bonding, there was no significant difference in percentage decrease of HD among the treatment groups (*P* > 0.05). After 14 days, CHI and CHT groups had greater percentage decrease of HD than the control (*P* < 0.001 and *P* = 0.009, respectively). Under SEM-EDS analysis, acid-etched dentin with CHI presented opened dentinal tubule orifices and more chlorhexidine precipitates on dentin than the dentin with CHT, which strongly related to a higher percentage of chloride ions on the CHI dentin surface (*P* < 0.001).

**Conclusion:**

The use of CHI on acid-etched dentin had a positive effect on dentin sealing ability of etch-and-rinse adhesive.

## Introduction

In contemporary restorative dentistry, the dental adhesive systems have been developed to provide the clinical success of tooth restoration including good bond strength and excellent dentin sealing ability.^1^ As dentin is biological mineralized tissue, it consists of hydroxyapatite crystallites embedded in collagen-based extracellular matrix.^2^ In etch-and-rinse adhesive system, the hybrid layer, or the bond between adhesive monomers and the exposed dentin matrix, should be preserved for a good dentin bonding stability.^3^^,^^4^ Previous studies reported that endogenous matrix metalloproteinases were the crucial factors of the degradation of the incompletely infiltrated collagen fibrils in the hybrid layer, and the degradation of hybrid layer might decrease the durability of resin-dentin bonds.^3^, ^4^, ^5^

However, the use of chlorhexidine on acid-etched dentin during bonding procedures of the etch-and-rinse adhesive could inhibit the activity of matrix metalloproteinases (MMP-2, MMP-8 and MMP-9),^6^^,^^7^ and preserved the resin-dentin bonding integrity both *in vitro*^8^, ^9^, ^10^ and *in vivo* studies.^11^^,^^12^ Furthermore, chlorhexidine has a good ability to bind to demineralized dentin more than eight fold in comparison to the mineralized one,^13^ and it belongs to the rehydration capacity to re-expand collagen fibrils of etched dentin.^14^ Thus, chlorhexidine is recommended to be applied on the acid-etched dentin without rinsing^15^ as a MMP inhibitory agent with a beneficial effect on the longevity of the hybrid layer and dentin bond strength.^16^

In addition, chlorhexidine is widely used as a cavity disinfectant prior to restorative procedures to reduce the number of cariogenic bacteria, due to its antibacterial action.^17^^,^^18^ However, the use of passive chlorhexidine treatment (CHT) could not complete the elimination of residual intratubular bacteria in coronal dentin.^18^, ^19^, ^20^ Thus, an increase in penetration depth of chlorhexidine irrigant into dentinal tubules using iontophoresis may be useful for the better cleaning efficacy in the tubules, as previous studies used iontophoresis to enhance drug delivery through human dentin for treating dentin hypersensitivity and improving dentin sealing ability of dental adhesives.^21^^,^^22^ Additionally, Ren et al. demonstrated that anode iontophoresis could enhance the delivery of chlorhexidine into and across bovine palate and gingiva, and might be an effective method to improve the treatment of periodontal disease.^23^ However, there was no study focusing on the effect of iontophoresis on the permeation of chlorhexidine into the dentin surface. Thus, it would be of great interest to study the effect of anode iontophoresis on the delivery of chlorhexidine into human dentin.

Besides antimicrobial activity, chlorhexidine had the MMP inhibitory effect and played an important role on the preservation of resin-dentin bonds when used with the etch-and-rinse adhesives.^15^ Although the application of low concentration of chlorhexidine for a short period of 15 s was sufficient for the preservation of the resin-dentin bonds,^24^ the use of 2% chlorhexidine for 60 s revealed the highest micro-tensile bond strength and the most dense dentin hybrid layer, when compared to the other MMP inhibitors.^25^ Furthermore, it was found that the application of 2% chlorhexidine for 15 s led to a decrease in the micro-shear bond strength of Single Bond 2 adhesive after 15-day storage in distilled water.^26^ However, there were no studies that focus on the effect of CHI in human dentin and its effect on dentin sealing ability of etch-and-rinse adhesive. The dentin sealing ability of adhesive is important to provide a tight seal against the microleakage and preserve the health of pulpodentin complex.^1^ The bonding durability ensures that the sealing of dentinal tubules is maintained.^27^ Good dentin sealing ability provided by dental adhesive is related to a decrease in hydraulic conductance (HD) of hybridized dentin as it against the fluid pass through the resin-dentin interface.^21^

Therefore, the objective of the study was to determine the effect of 2% CHI for 60 s as a pretreatment of acid-etched dentin on dentin sealing ability of etch-and-rinse adhesive in human extracted teeth after immediate bonding and after 14 days using HD measurement and SEM-EDS analysis. The tested null hypothesis was that the 2% CHI would have no effect on percentage decrease of HD of etch-and-rinse adhesive after bonding and after 14 days, when compared to the CHT and control.

## Materials and methods

The present study was approved by the Institutional Review Board, Faculty of Dentistry/Faculty of Pharmacy, Mahidol University, Thailand (COE.No.MU-DT/PY-IRB 2021/021.2106), and performed in accordance with the Declaration of Helsinki. As the study used the extracted teeth in such a manner that subjects could not be identified from the beginning by code (unidentifiable data), the need for informed consent was waived by the Institutional Review Board, Faculty of Dentistry/Faculty of Pharmacy, Mahidol University, Thailand (COE.No.MU-DT/PY-IRB 2021/021.2106). Thirty-nine human intact third molars were obtained from Oral and Maxillofacial Surgery Clinic, Dental Hospital, Faculty of Dentistry, Mahidol University. The teeth were extracted for dental reasons, including prophylactic removal, eruption problems, and orthodontic purposes. All extracted teeth were kept in 0.1% thymol solution (M Dent, Nakhon Pathom, Thailand), and used within 2 weeks. The teeth with crack, craze line, dental caries, fillings, or crown restoration were excluded.

### Tooth preparation

Thirty-nine sound dentin specimens were prepared from 39 extracted intact third molars. Each intact tooth was sectioned 3 mm below and above the cemento-enamel junction using a diamond blade (NTI, Kahla, Germany) with water coolant until occlusal dentin was exposed. To obtain a standardized smear layer, the occlusal dentin surface was abraded under water-cooling with 600 grit silicon carbide abrasive paper for 15 s. For each crown specimen, pulpal tissue remnant was removed with tweezers, and pulpal cavity was rinsed with distilled water for 30 s. The remaining dentin thickness (RDT) was controlled using Iwanson caliper, item no. 4733 (Kohler, Stockach, Germany) which measured the RDT1 between the flat occlusal dentin surface and the highest pulp horn, and the RDT2 between the occlusal surface and the thickest of dentin in the middle of the roof of the pulp chamber. The mean RDT1 and RDT2 of dentin specimens of 3 different treatment groups were calculated and statistically compared using one-way ANOVA test to confirm that there was no significant difference in the mean values among the treatment groups (Table 1) to minimize the influence of variation in dentin depths on dentin permeability and dentin sealing ability. After that, the specimen was attached to an acrylic block with cyanoacrylate (Koatsu Gas Kogyo Co. Ltd., Osaka, Japan), and connected to the HD measuring device.^21^

**Table 1:** The means and standard deviations (SD) of remaining dentin thickness (RDT) of dentin specimens of different treatment groups.

RDT (mm)	Control	CHT	CHI	1-way ANOVA
RDT1	1.99 (0.40)	1.87 (0.54)	2.09 (0.53)	*P* = 0.615
RDT2	2.90 (0.39)	3.07 (0.66)	3.05 (0.41)	*P* = 0.715

There were no significant differences among the different treatment groups regarding the RDT1 (*P* = 0.615) and the RDT2 (*P* = 0.715). Abbreviations: CHT: passive chlorhexidine treatment; CHI: chlorhexidine iontophoresis; RDT1: RDT between the occlusal surface and the highest pulpal horn; RDT2: RDT between the occlusal surface and the thickest of dentin in the middle of the roof of the pulp chamber.

### Hydraulic conductance measurement

Thirty specimens were randomly selected for HD measurement. The occlusal dentin surfaces were subjected to bonding with etch-and-rinse adhesive (Adper™ Single Bond 2; 3M ESPE, St. Paul, MN, USA). Table 2 shows chemical compositions and manufacturer's instructions of the etch-and-rinse adhesive system used in the study. HD of each specimen was measured before treatment, after immediate bonding and after 14 days.

**Table 2:** Chemical compositions and manufacturer's instructions of the etch-and-rinse adhesive system used in the study.

Material used (batch number)	Manufacturer	Composition	Manufacturer's instruction
Scotchbond etchant gel (NC56097)	3M ESPE, St. Paul, MN, USA	35% phosphoric acid by weight (pH 0.6), fumed silica, water soluble surfactant	Etchant: Apply etchant for 15 s. Rinse for 10 s.
Adper single bond 2 (NA89171)	3M ESPE, St. Paul, MN, USA	Bis-GMA, HEMA, dimethacrylates, ethanol, water, photoinitiator, methacrylate functional copolymer of polyacrylic and polyitaconic acids, 10% 5-nm-diameter spherical silica particles	Adhesive: Immediately after blotting, apply 2–3 consecutive coats of adhesive to etched dentin for 15 s with gentle agitation using a fully saturated applicator. Gently air thin for 5 s to evaporate solvents. Light cure for 10 s.

Abbreviations: Bis-GMA: Bisphenol glycidyl methacrylate; HEMA: 2-hydroxyethyl methacrylate.

**Figure 1:**
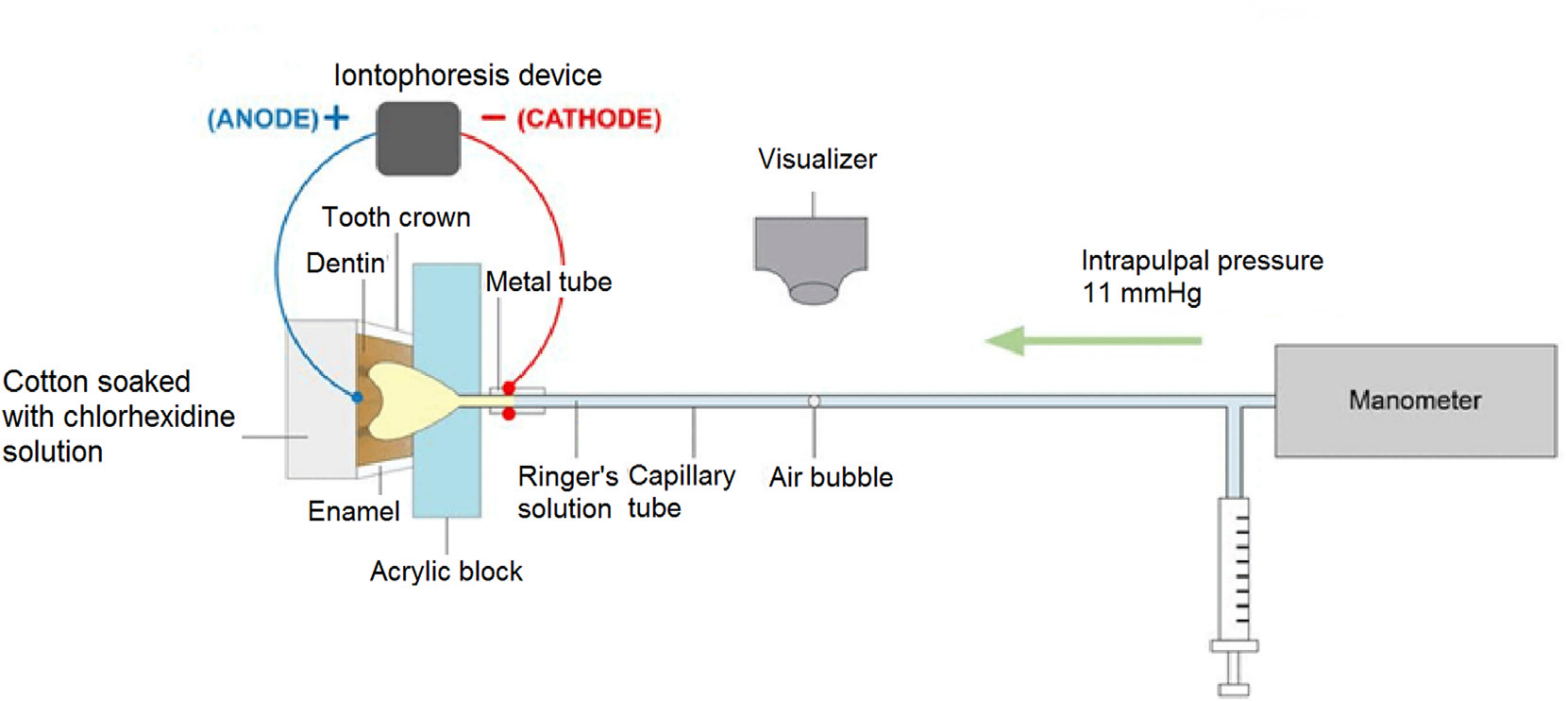
Diagram of the experimental set up for chlorhexidine iontophoresis.

After tooth preparation, the exposed occlusal dentin was covered with cotton soaked with Ringer's solution to prevent dentin dehydration, to make an isotonic condition similar to dentinal fluid, and to stabilize the physiological pH (at pH = 7.4), as dentinal fluid in vital dentin continuously filled with the pulpal interstitial fluid under a positive pulp pressure and its electrolyte content similar to that of plasma.^2^ Also, the fluid system in the HD measuring device was filled with Ringer's solution.^28^ Baseline HD of dentin with smear layer (before treatment) was measured by observing the movement of a small air bubble which introduced into a capillary using visualizer and the pressure in the fluid filtration system was set at 100 mmHg above atmospheric using a mercury manometer to represent hydrostatic pressure stimulus which increased dentin sensitivity to outward flow.^28^ Thereafter, each occlusal dentin surface was etched with 35% phosphoric acid (Scotchbond™ Etchant gel; 3M ESPE, St. Paul, MN, USA) for 15 s. Then, acid-etched dentin surface was rinsed with distilled water for 10 s. The excess water was removed using absorbent paper until the dentin surface appeared glistening without pooling of water. After acid etching, 30 specimens were randomly divided into 3 groups;

Group 1: No chlorhexidine treatment group (control, n = 10).
Group 2: CHT group (n = 10). 2% chlorhexidine gluconate solution (M Dent, Bangkok, Thailand) was applied with a micro applicator (Cotisen, Hebei, China),^25^^,^^26^ and left on the acid-etched dentin surface for 60 s. The excess solution was removed using absorbent paper.
Group 3: CHI group (n = 10). The occlusal dentin covered with cotton soaked with 2% chlorhexidine gluconate solution (M Dent). Then, the tooth specimen was connected with iontophoretic applicator (Iontophor II Model 6111 PM/DX; Life-Tech Inc, Stafford, TX, USA) for applying anode electric current at 0.5 mA for 60 s under intrapulpal pressure of 11 mmHg, representing normal tissue pressure in the dental pulp (Fig. 1).^29^ After that, the fluid excess was removed using absorbent paper.

Immediately after blotting, the occlusal dentin surfaces of all dentin specimens were applied with 2–3 consecutive coats of adhesive (3M ESPE) for 15 s with gentle agitation using a fully saturated applicator (Cotisen), and the solvent was evaporated by gently air thin for 5 s. Then, the adhesive-dentin surfaces were light-cured with a halogen lamp-curing unit (3M ESPE Elipar ™ 2500; 3M ESPE, St. Paul, MN, USA) for 10 s in accordance with the manufacturer's instructions. The HD of bonded dentin (after immediate bonding) was measured again as described above. After that, the specimens were immersed in Ringer's solution and kept in the temperature controlled chamber (Memmert, Schwabach, Germany) at 37 °C for 14 days. The storage media was changed on a weekly basis. Thereafter, HD of bonded dentin after 14 days was re-measured again. To compensate for the large variations in permeability of the different dentin specimens, the HD measurements made on the same dentin specimen were expressed as a percentage increase or decrease relative to the baseline HD of dentin with smear layer. Thus, each specimen served as its own control.

For each specimen, the percentage (%) decrease of HD through the adhesive-dentin interface or dentin sealing ability of adhesive after immediate bonding and after 14 days were calculated by the following formula: the % decrease of HD (after bonding/after 14 days) = {[HD (before treatment) − HD (after bonding/after 14 days)]/HD (before treatment)} × 100%.

### Scanning electron microscopy and energy dispersive x-ray spectroscopy

The other 9 sound dentin specimens were prepared and randomly divided into 3 equal-sized groups: acid-etched dentin, acid-etched dentin with CHT, and acid-etched dentin with CHI for SEM-EDS study, using a scanning electron microscope (JSM-6610 LV; JEOL, Tokyo, Japan) and energy dispersive x-ray spectrometer with an X-Max 20 mm^2^ detector (Oxford Instruments Inc., High Wycombe, UK). Each specimen was observed to assess the characteristics of acid-etched dentin and chlorhexidine precipitation on dentinal surface. In addition, the dispersive spectroscopic analysis of each specimen was performed at 2 separate locations on each dentin surface to determine elemental composition (n = 6/group). As dentin is mineralized tissue which contains both hydroxyapatites and collagen fibrils, the peaks of P, Ca and O indicate the presence of the calcium hydroxyapatites, and the peaks of C, N and O indicate the components of collagen fibrils.^30^ C, N and Cl are the constituents of chlorhexidine [−(CH_2_)_3_ NHC (=NH) NHC (=NH) NHC_6_H_4_Cl]_2_. The peak of Cl serves as a marker of chlorhexidine deposits in the dentin specimens.^31^^,^^32^ Therefore, in the chemical analysis by EDS, the following elements: C–carbon, N–nitrogen, O–oxygen, P–phosphorus, Cl–chloride and Ca–calcium were determined. The calcium and phosphorus (Ca/P) ratios were calculated using the following formula, taking into account the respective atomic masses:

Ca(mol)/P(mol) = [Ca(weight%)/40.08(g/mol)]/[P(weight%)/30.97(g/mol)].

### Statistical analysis

The percentage decreases of HD, the weight% and atomic% of each element and the Ca/P ratio of dentin were represented as mean ± standard deviation (SD). The data were analyzed using the SigmaPlot 11.0 (Systat Software Inc.; San Jose, CA, USA). The Shapiro–Wilk test was performed to test the normal distribution of the data. Regarding the different treatment groups and observation periods, the mean percentage decreases of HD were compared using two-way repeated measures analysis of variance (2-way RM ANOVA). Where this showed a significant effect, Student-Newman–Keuls method (SNK method) was used for pairwise multiple comparisons.

For analysis of the weight% and atomic% of each element and the Ca/P ratio of dentin, the means of different treatment groups were compared using one-way analysis of variance (1-way ANOVA). Where this showed a significant effect, Student-Newman–Keuls method was used for pairwise multiple comparisons.

*P* values less than 0.05 were considered significant.

## Results

### Percentage decrease of hydraulic conductance of dentin

**Figure 2:**
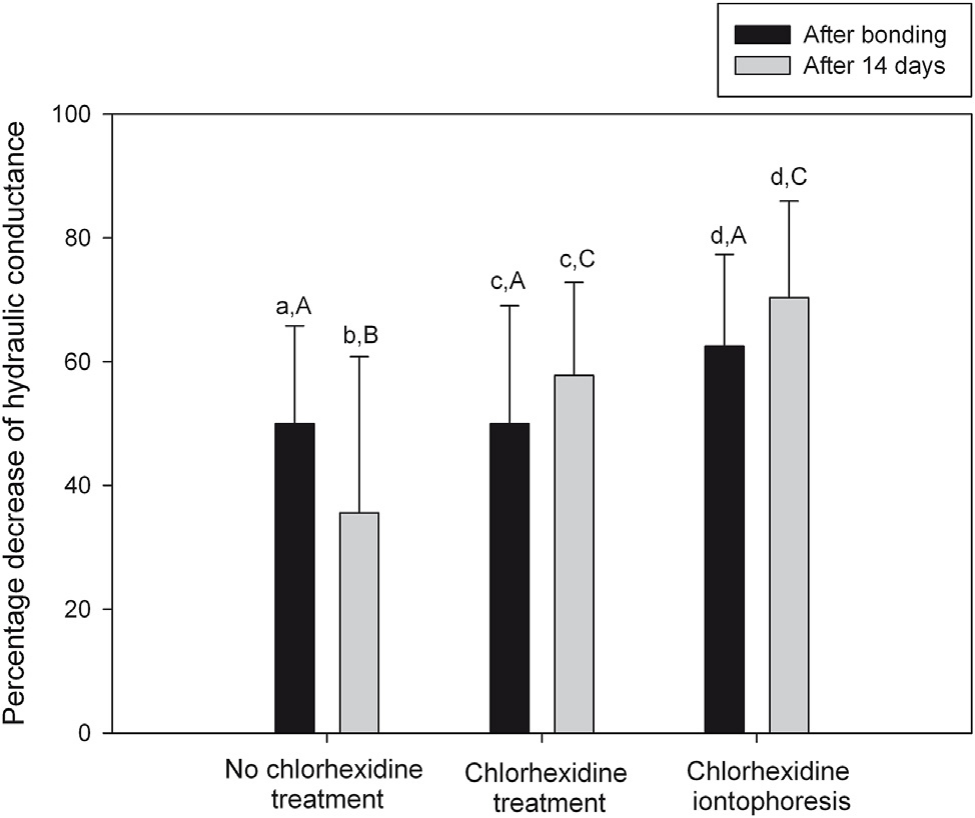
Mean (±1 SD) percentage decreases of hydraulic conductance of human dentin after bonding immediately (black column) and after 14 days (gray column) in no chlorhexidine treatment, passive chlorhexidine treatment and chlorhexidine iontophoresis groups. The same lowercase letter represents no significant difference between after bonding and after 14 days. The same uppercase letter represents no significant difference among the treatment groups (*P* > 0.05, 2-way RM ANOVA and Student-Newman-Keuls method).

The means and SDs of percentage decrease of HD of dentin after immediate bonding and after 14 days in each group are shown in Fig. 2. Two-way RM ANOVA revealed that there was a statistically significant interaction between the treatment factor and the observation period factor (*P* = 0.001). The effect of different treatments depended upon what observation period was present. In control group, HD reduced by 50.01 ± 15.78% after bonding and 35.59 ± 25.23% after 14 days, with a significant difference between observation periods (*P* = 0.003). In CHT group, HD reduced by 50.01 ± 19.03% after bonding and 57.76 ± 15.04% after 14 days. In CHI group, HD reduced by 62.49 ± 14.86% after bonding and 70.35 ± 15.58% after 14 days. Both CHT and CHI groups showed no significant difference among the observation periods (*P* = 0.090 and *P* = 0.085, respectively). In comparison among the treatment groups, there was no significant difference in the mean values after bonding (*P* > 0.05). However, after 14 days, CHT and CHI groups had greater percentage decrease of HD than the control (*P* = 0.009, *P* < 0.001, respectively).

### Scanning electron microscopy and energy dispersive x-ray spectroscopy findings

**Figure 3:**
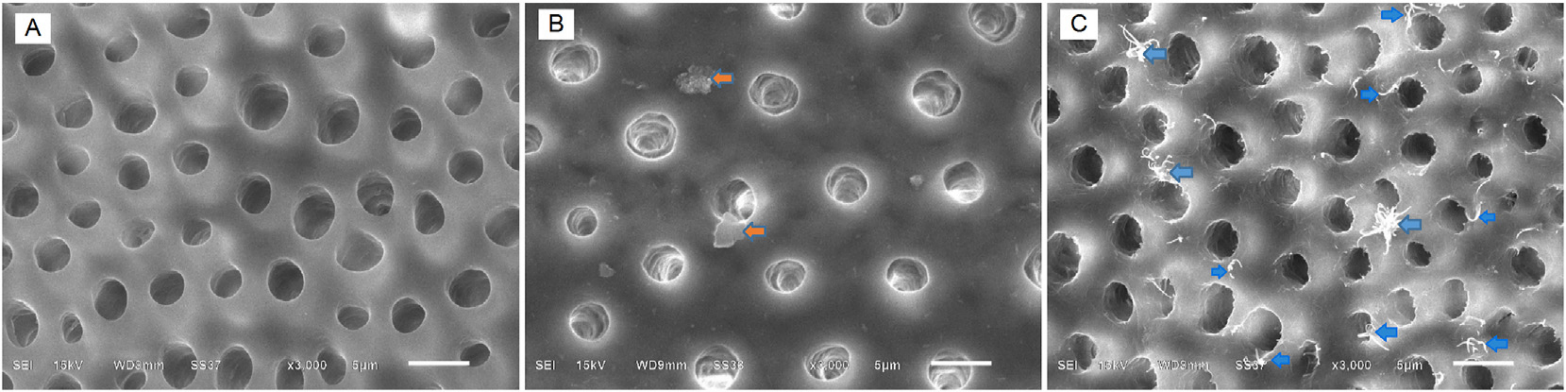
(A–C) Scanning electron micrographs of etched dentin surface (original magnification X3000, scale bar = 5 μm): (A) no chlorhexidine treatment (control); (B) passive chlorhexidine treatment; (C) chlorhexidine iontophoresis. Arrow heads showing chlorhexidine precipitates on treated dentin surfaces.

The dentin surface of acid-etched dentin was smooth, smear layer and smear plugs were removed and the dentinal tubule orifices were visible (Fig. 3A). In group of acid-etched dentin with CHT, the opened tubule orifices remained visible and chlorhexidine precipitates deposited loosely on the dentin surfaces (Fig. 3B). The precipitates were described as crystal cluster micrometric-sized structures (orange arrows). In group of acid-etched dentin with CHI, opened dentinal tubules and more numerous chlorhexidine precipitates could be observed (Fig. 3C). The precipitates were described as dendritic crystal cluster forming structures (blue arrows).

**Figure 4:**
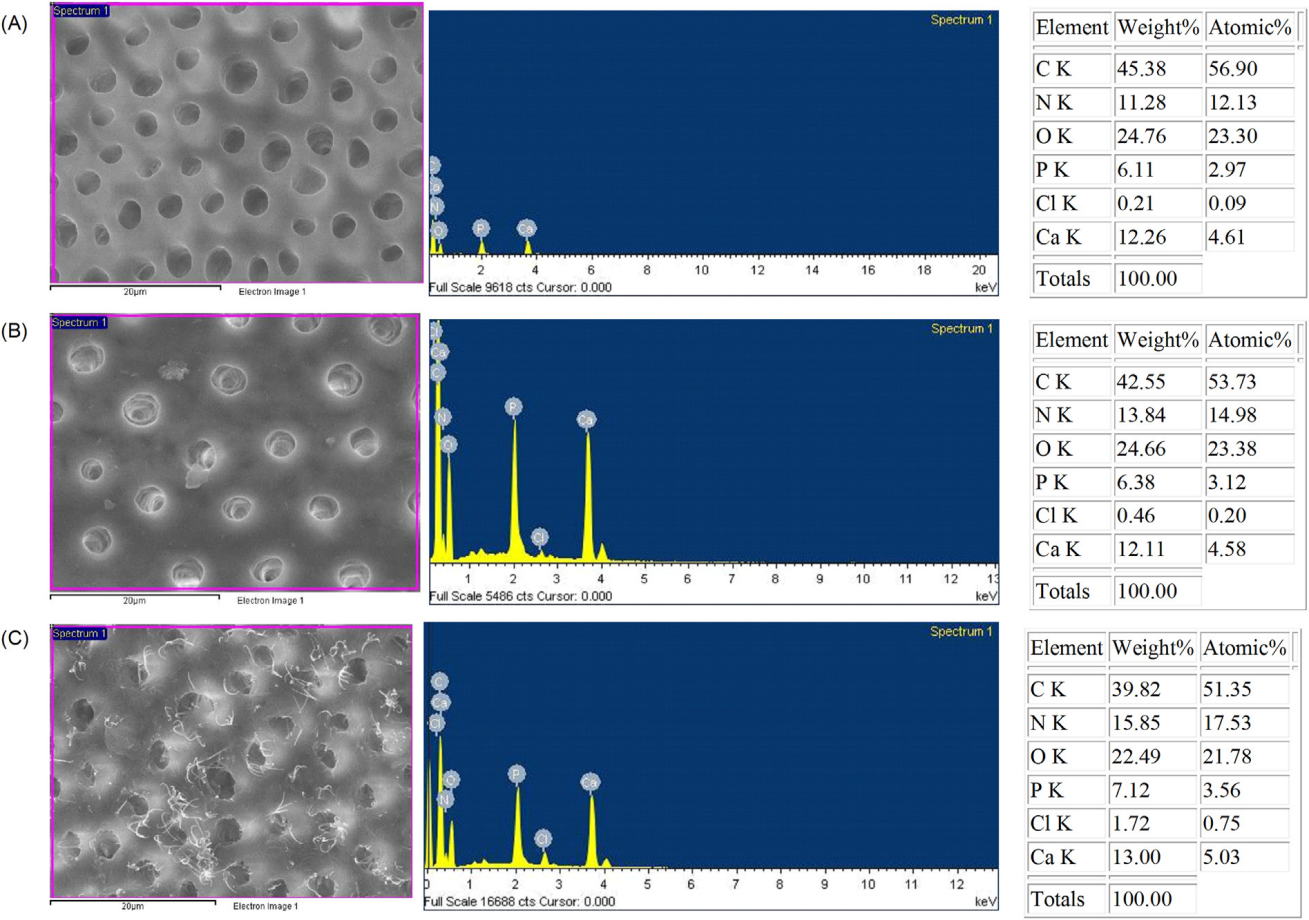
Energy-dispersive X-ray spectroscopy (EDS) spectra taken from the treated dentin surface of (A) No chorhexidine treatment, (B) passive chlorhexidine treatment, (C) chlorhexidine iontophoresis with the right table for the weight and atomic percentage of the following elements: C–carbon, N–nitrogen, O–oxygen, P–phosphorus, Cl–chloride, Ca–calcium.

Elemental composition of these dentin specimens is presented in Table 3. The example of EDS-spectrum taken from each treated dentin surface is presented in Fig. 4. As acid-etched dentin had a very small amount of %Cl, the addition of 2% chlorhexidine to dentin could cause a significant change in the weight% and atomic% of Cl. Strong Cl signal was identified after chlorhexidine treatment (Fig. 4B), and increased amount of Cl was detected in the dentin with CHI (Fig. 4C). CHT and CHI groups had greater weight% and atomic% of Cl than the control (for weight%: *P* = 0.031, *P* < 0.001, respectively; for atomic%: *P* = 0.027, *P* < 0.001, respectively). However, there was not a statistically significant difference in the means of Ca/P ratio among the treatment groups (*P* = 0.977).

**Table 3:** The elemental analysis of 3 different treated dentin groups in terms of weight% and atomic% of elements (C–carbon, N–nitrogen, O–oxygen, P–phosphorus, Cl–chloride, Ca–calcium) and the calcium and phosphorus (Ca/P) ratio. The mean values and standard deviations (SD) are shown and taken from 6 different areas of the 3 specimens per each treated group.

Element	Weight%			Atomic%		
	Control	CHT	CHI	Control	CHT	CHI
C	46.77 (4.12)^A^	41.00 (1.54)^B^	42.82 (2.91)^B^	56.52 (2.79)^a^	52.10 (1.39)^b^	53.65 (2.35)^b^
N	14.66 (1.73)^A^	14.10 (0.94)^A^	15.40 (0.93)^A^	15.21 (1.59)^a^	15.36 (0.92)^a^	16.56 (0.89)^a^
O	25.07 (1.12)^A^	25.57 (0.48)^A^	23.58 (1.51)^B^	22.79 (0.81)^a^	24.40 (0.69)^b^	22.21 (1.61)^a^
P	4.54 (1.77)^A^	6.50 (0.49)^B^	5.88 (0.96)^B^	2.16 (0.92)^a^	3.21 (0.29)^b^	2.87 (0.54)^b^
Cl	0.21 (0.03)^A^	0.50 (0.08)^B^	1.23 (0.35)^C^	0.09 (0.01)^a^	0.22 (0.03)^b^	0.52 (0.15)^c^
Ca	8.75 (3.96)^A^	12.33 (1.09)^A^	11.09 (2.04)^A^	3.23 (1.57)^a^	4.70 (0.48)^a^	4.18 (0.87)^a^
Ca/P ratio	1.45 (0.13)^A^	1.46 (0.03)^A^	1.45 (0.07)^A^	1.45 (0.13)^a^	1.46 (0.03)^a^	1.45 (0.07)^a^

For each element, the same uppercase and lowercase letter represent no significant difference among the treatment groups in terms of weight% and atomic%, respectively (*P* > 0.05, 1-way ANOVA and Student-Newman-Keuls method). Abbreviations: CHT: passive chlorhexidine treatment; CHI: chlorhexidine iontophoresis.

## Discussion

This study demonstrated that after 14 days, CHI and CHT groups had better dentin sealing ability than the control group. Chlorhexidine still remained on the acid-etched dentin surfaces after chlorhexidine treatment. The dentin with CHI had more numerous chlorhexidine deposits, which strongly related to a higher percentage of Cl ions on the dentin surface, when compared to the dentin with CHT. Therefore, the null hypothesis was rejected.

Iontophoresis is one of the widely used non-invasive drug delivery techniques for promoting ionized molecules, unionized molecules, and high molecular weight compounds to transport through tissue barriers.^23^^,^^33^ Although many previous studies used iontophoresis to enhance drug delivery through dentin for treating dentin hypersensitivity, producing local anesthesia, or improving dentin sealing ability of dental adhesives,^21^^,^^22^^,^^33^ the present study is the first to demonstrate the use of CHI in human dentin. As chlorhexidine is positively charged, anodal iontophoresis can be used following the principle that positively charged drug ions (cations) are repelled by a positive electrode (anode) and run to a negative electrode (cathode) for repelling chlorhexidine into tissues.^23^ Likewise, anode iontophoresis could augment the permeation of positively charged chlorhexidine on the acid etched dentin. Therefore, the present study found that CHI could enhance more chlorhexidine binding to dentin than the CHT. The dentin with CHI had more numerous chlorhexidine deposits, which strongly related to a significantly higher percentage of Cl ions on the dentin surface than the dentin with CHT. Although there was the statistical insignificance of the percentage decrease of HD between CHT and CHI, the both groups gave better dentin sealing ability after 14 days than the control.

In etch-and-rinse adhesive system, the hybrid layer at the adhesive-dentin interface is the major bonding mechanism which is responsible for the dentin sealing ability of adhesive.^1^ In relation to the dentin permeability, the adhesives with excellent dentin sealing ability could exhibit the less fluid flow through the adhesive-dentin interface, or lower the HD.^34^ The HD measurement is a non-destructive method, allowing the test to be performed in near-to-physiological circumstances with the simulated pulpal pressure and the same specimen to be measured repeatedly, and has been used to assess changes at the surface of dentin before and after application of dental adhesives.^21^^,^^27^ The present study found that the control group (no chlorhexidine treatment) had a decrease in dentin sealing ability of etch-and-rinse adhesive after 14 day-storage, reduced from 50.01% after immediate bonding to 35.59% after 14 days. In agreement with the study of Cantahede de Sa et al., the dentin sealing ability of adhesives was reduced after aging because phosphoric acid etching procedure in the bonding protocol could activate the host-derived matrix metalloproteinases (MMPs) in demineralized dentin, which led to the degradation of hybrid layer and increased dentin permeability.^27^ Additionally, Mazzoni et al. reported that phosphoric acid etching with etch-and-rinse adhesive increased MMPs levels about 3.5 times when compared with phosphoric etching alone.^35^ The presence of MMPs was the crucial factor of the degradation of resin-dentin bonds, and caused a decrease in dentin sealing ability overtime.^4^^,^^27^ Therefore, the preservation of hybrid layer by using MMP inhibitors such as 2% chlorhexidine could help to prolong the bond durability of etch-and-rinse adhesives.^16^ In agreement with the literature, our study found that both CHI and CHT for 60 s resulted in a similar dentin sealing ability after 14 days to the sealing ability after immediate bonding. Whereas, Silva et al. reported that CHT for a short period of 15 s had a decrease in the bond strength of Single Bond 2 adhesive after 15-day storage in distilled water.^26^ However, in their study, the use of CHT in the bonding protocol increased the bond strength when compared to the bonding protocol without CHT.^26^

Chlorhexidine is a synthetic cationic molecule consisting of the two symmetric structures with 4 chlorophenyl rings and 2 biguanide groups connected by a central hexamethylene bridge.^36^ After chlorhexidine treatment on acid-etched dentin, the positive charges of chlorhexidine can bind electrostatically to the negatively charged carboxyl groups on collagen of demineralized dentin, and cationic chlorhexidine can also hydrogen bond with carboxyl groups in the collagen peptides.^13^ In agreement with the study of Lapinska et al., chlorhexidine-treated acid-etched dentin presented open orifices of dentinal tubules and chlorhexidine deposits on the dentinal surface could be observed.^32^ Furthermore, due to the substantivity of chlorhexidine to human dentin, chlorhexidine still remained active in human dentin for a prolong period,^36^ and served as an MMP inhibitor for the long-term bond stability of etch-and-rinse adhesive system.^16^ Previous studies reported that pretreatment of dentin with chlorhexidine solution did not affect the immediate bond strength and nanoleakage of dentin, and it also helped to preserve bond strength and lower the nanoleakage overtime from 6 months to 10 years.^8^, ^9^, ^10^, ^11^, ^12^ Therefore, in agreement with the previous studies, both CHI and CHT groups presented better dentin sealing ability of adhesive than the control group after 14 days of storage.

Consistent with the SEM study, after an application of 2% chlorhexidine solution either with or without iontophoresis, the presence of chlorhexidine precipitates was observed on the treated dentin surface as crystal clusters. This occurrence might be due to the binding of two positive charges of chlorhexidine and the negative charges of trivalent phosphate groups in hydroxyapatite crystals.^32^^,^^37^ In addition, using elemental analysis, the present study revealed no loss of calcium and no change in the calcium/phosphorus ratio after chlorhexidine treatment either with or without iontophoresis. Furthermore, Perdigao et al. reported that the chlorhexidine precipitates on the etched dentin surface did not reduce the bond strength of etch-and-rinse adhesive system.^37^ Therefore, the presence of chlorhexidine precipitates did not interfere with the adhesion of etch-and-rinse adhesive to dentin, and it might help to improve the bonding area for the better dentin sealing ability of adhesive. Moreover, 2% CHI for 60 s produced more chlorhexidine deposits and still reduced the HD of dentin. Hence, it would suggest that the use of iontophoresis with chlorhexidine at a shorter contact time (30 s or less) may be more clinically acceptable than the passive treatment. Besides that, anode iontophoresis can be used to enhance the penetration of chlorhexidine in human dentin (e.g., for dentin disinfection prior to adhesive treatment) with no negative effect on the dentin sealing ability of etch-and-rinse adhesive.

Within the limitation of this study, further studies are necessary to evaluate its effect in the presence of caries affected dentin. As the caries affected dentin lowered the ability of drug diffusion during iontophoresis, and it might affect the dentin sealing ability of dental adhesive.^21^ Also, the long term effect and the *in vivo* studies should be observed. Additionally, regarding the limitation of this study, the penetration depth of 2% chlorhexidine solution into coronal dentinal tubules could not be determined. However, a previous study using confocal laser scanning microscope reported that the penetration depth of 2% chlorhexidine into coronal third of root dentin was about 138 μm when root canal was irrigated with convention syringe for 3 min.^38^ Further studies about the effect of iontophoresis on permeation of 2% chlorhexidine into human dentin are needed to be stronger.

In summary, good dentin sealing ability of dental adhesive is related to a decrease in HD of adhesive-dentin interface as it resists the fluid pass through the surface. In the study, the etch-and-rinse adhesive was able to reduce the HD of the prepared dentin after immediate bonding. However, after 14 days, the control (no chlorhexidine treatment) had a decrease in dentin sealing ability of etch-and-rinse adhesive, meanwhile, the use of CHI or CHT for 60 s in the bonding protocol had better dentin sealing ability of adhesive than the control group. Thus, the use of CHI on acid-etched dentin could enhance the deposit of chlorhexidine in human dentin, and had a positive effect on dentin sealing ability of etch-and-rinse adhesive.

## Declaration of competing interest

The authors have no conflicts of interest relevant to this article.
